# Scaling Up Malaria Control in Zambia: Progress and Impact 2005–2008

**DOI:** 10.4269/ajtmh.2010.10-0035

**Published:** 2010-09

**Authors:** Elizabeth Chizema-Kawesha, John M. Miller, Richard W. Steketee, Victor M. Mukonka, Chilandu Mukuka, Abdirahman D. Mohamed, Simon K. Miti, Carlos C. Campbell

**Affiliations:** National Malaria Control Center, Ministry of Health, Ndeke House, Lusaka, Zambia; Malaria Control and Evaluation Partnership in Africa (MACEPA), PATH, Lusaka, Zambia; Ferney-Voltaire, France, and Seattle, Washington; Ministry of Health, Ndeke House, Lusaka, Zambia

## Abstract

Zambia national survey, administrative, health facility, and special study data were used to assess progress and impact in national malaria control between 2000 and 2008. Zambia malaria financial support expanded from US$9 million in 2003 to US$ ~40 million in 2008. High malaria prevention coverage was achieved and extended to poor and rural areas. Increasing coverage was consistent in time and location with reductions in child (age 6–59 months) parasitemia and severe anemia (53% and 68% reductions, respectively, from 2006 to 2008) and with lower post-neonatal infant and 1–4 years of age child mortality (38% and 36% reductions between 2001/2 and 2007 survey estimates). Zambia has dramatically reduced malaria transmission, disease, and child mortality burden through rapid national scale-up of effective interventions. Sustained progress toward malaria elimination will require maintaining high prevention coverage and further reducing transmission by actively searching for and treating infected people who harbor malaria parasites.

## Background

With the launch of the Roll Back Malaria (RBM) Partnership in 1998, global malaria control partners set out to “halve the burden of malaria by 2010,” by reducing malaria-associated morbidity and mortality by that amount.^1^ In Abuja, Nigeria, the partners committed to achieving 60% coverage with preventive interventions among those at risk of malaria and 60% coverage with prompt and effective treatment among those suffering from malaria.^2^ With the development of the 2005–2015 RBM Strategic Plan, the targets were raised to 80% and further specified for the interval to 2010 and to 2015, including achieving the Millennium Development Goals (MDGs) and equitable coverage.^3^

In November 2007, malaria partners reset ambitious goals to include elimination of malaria in some countries as defined in the Global Malaria Action Plan of 2008.^4^ Although the targets for malaria control have changed rapidly in the past decade, at the technical level of monitoring and evaluation, the measures have adhered to the standard monitoring and evaluation (M&E) framework.^5^

Here, the national malaria control program effort in Zambia is reviewed as an example of a fully malaria-endemic country that achieved rapid success from 2005 through 2008 and is moving to sustain that success and consider next steps to advance its control of the malaria burden and transmission. The progress and components of success to date are described, as are the evolving requirements for success as the program proceeds.

### Country background: Zambia.

Zambia has a population of approximately 12 million people in 9 provinces and 72 districts and is fully malaria endemic with regular and moderate to high transmission in all districts and with a seasonal pattern of high transmission between December and May associated with the rains.^6^ Zambia's initial National Malaria Control Strategic Plan covered the period from 2000 to 2005; the plan was updated for 2006 to 2010, setting ambitious goals to scale up a package of malaria interventions: insecticide-treated mosquito nets (ITNs), indoor residual spraying (IRS); prevention with intermittent preventive treatment in pregnancy (IPTp) and ITNs; and case management with diagnosis using microscopy and rapid diagnostic tests (RDTs) and prompt effective treatment with artemisinin-based combination therapy (ACT) using artemether-lumefantrine (AL); see Table 1. Detailed information on Zambia malaria and malaria control efforts during the past decade can be found elsewhere.^7^,^8^

## Methods and Data Sources

Available information was reviewed from national surveys, program data, special studies, and in-country reports to assess malaria control progress in Zambia. After the standard monitoring and evaluation framework for RBM,^5^ authors examined policy and strategy decision making, the use of financial resources (inputs), service delivery (process and outputs), intervention coverage (outcomes), and health and economic improvements (impact) across urban and rural settings and across socioeconomic strata. Data available from the standard published reports, surveys, and special studies were reviewed and compared over time; no additional within-survey analyses were undertaken.

To ensure that available prevention and treatment interventions reach all Zambians, the Zambia National Malaria Control Center (NMCC) monitored commodity procurement and distribution. To assess progress toward achieving greater than 80% population coverage targets, several household surveys were considered, including the national UNICEF Multiple Indicator Cluster Survey (1999 MICS^9^), Demographic and Health Surveys (2001/2^10^ and 2007^11^-DHS), and Malaria Indicator Surveys (2006^12^ and 2008^13^-MIS), as well as the smaller-scale 2004 Roll Back Malaria (2004-RBM^14^) survey. These national surveys rely on asset-based indices to create relative proxy measure of household wealth.^15^ The NMCC also tracked progress using programmatic output information on service delivery for malaria interventions (e.g., for routine ITN and IRS delivery and operational coverage) and use of RDTs. Routine health management information systems (HMIS) reports from health facilities across the nation allowed for retrospective tracking of fever cases, malaria diagnosis, hospital admissions, and treatment activities^7^,^13^; however, because of recent modifications in the HMIS and some irregularities of reporting, the national evaluations here did not include the details of these data. Because environmental factors, particularly changes in rainfall patterns, can affect malaria transmission, we reviewed 12-month Weighted Anomaly Standardized Precipitation (WASP) data between 1980 and 2008 to assess possible affects of rainfall variation on malaria parasitemia.^16^

## Results

### Zambia's malaria control progress.

#### Sound policies attracting partners and growing resources.

**Figure 1.**
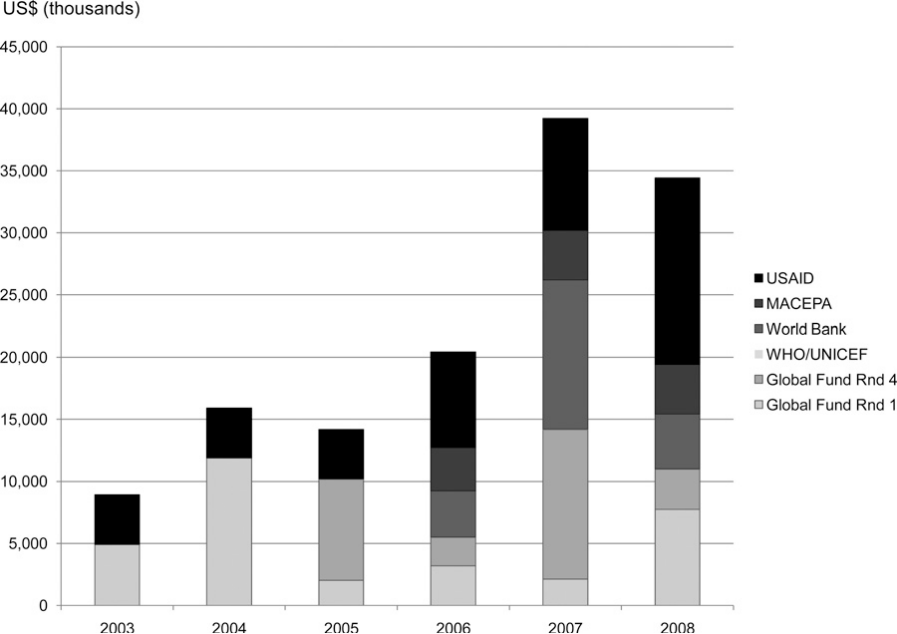
External funding support for Zambia malaria control: estimates for 2003–2008.

With a 2006–2010 national strategy for malaria control and consistent leadership from the Ministry of Health (MoH) and the NMCC, Zambia attracted new or increased funding from a variety of bilateral and multilateral sources. This began with the United States Agency for International Development (USAID) support during 2000–2003 and expanded greatly with Global Fund grants in Round 1, 4, and 7; support expanded further with funding from the World Bank, the Malaria Control and Evaluation Partnership in Africa (MACEPA, a program at PATH funded by the Bill & Melinda Gates Foundation) in 2005, and with the U.S. Presidents Malaria Initiative (PMI) in 2007; see Figure 1. Intermittent support from the Japanese International Cooperation Agency (JICA) and district health funding from multiple donors contributing to the Sector Wide Approach (SWAp) provided a variety of support needed for program action. Numerous other partners provided smaller financial contributions toward various aspects of technical assistance, advocacy, or service delivery at local level, including the World Health Organization (WHO) and UNICEF. The Government of the Republic of Zambia (GRZ) also has provided increasing resources from 2000 through 2008 for malaria control. In many respects, this amount is the most difficult to quantify specifically for malaria as the GRZ supports national staff providing the majority of services and the facilities (hospitals, health centers, health posts, laboratories, etc.) where malaria control and a range of other services are delivered.

#### Using funds and delivering services.

**Figure 2.**
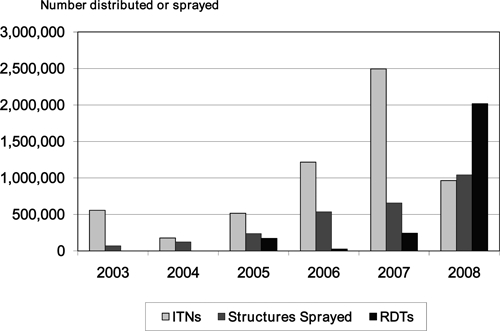
Number of insecticide-treated mosquito nets (ITNs) distributed structures sprayed and rapid diagnostic tests distributed by year in Zambia between 2003–2008.

**Figure 3.**
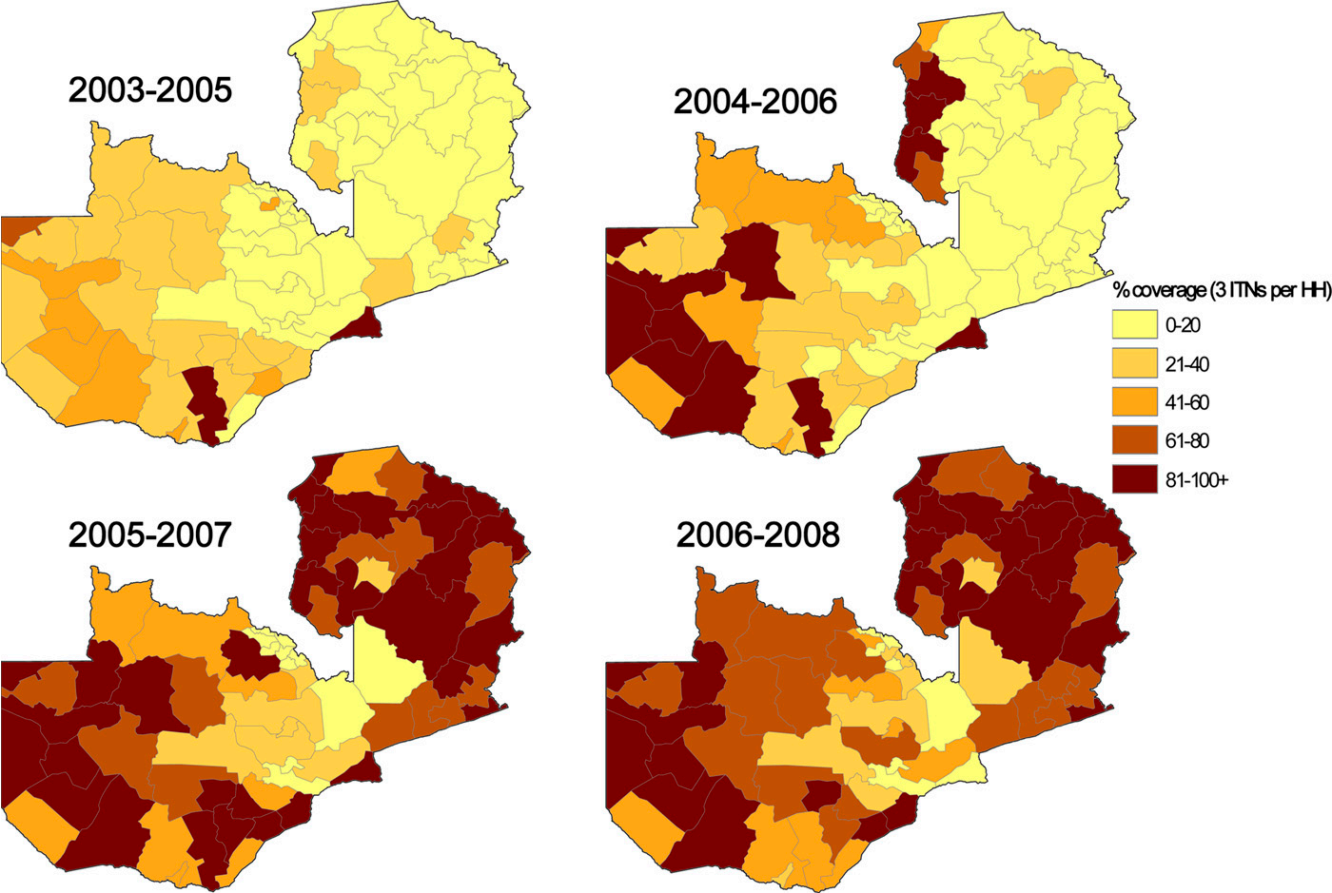
Estimated operational coverage of 3 insecticide-treated mosquito nets (ITNs) per household in overlapping 3-year intervals based on ITN distributions by district in Zambia during 2003–2008. This figure appears in color at www.ajtmh.org.

Malaria prevention and diagnostic services expanded markedly from 2003 through 2008. During this period, the Zambia Ministry of Health and partners procured and distributed approximately 5.9 million ITNs (since 2007 all are long-lasting or LLINs), nearly 80% of which were distributed under intensified efforts from 2006 through 2008, with the largest distribution occurring in 2007 and less in 2008 because most of the acute need was addressed in the previous year (Figure 2). District-based rolling mass distribution enabled wide coverage, especially to rural and previously poorly served areas (Figure 3). National IRS activities including mapping and enumerating IRS target areas, procurement of insecticide and spray equipment, and training of spray teams, expanded from 5 to 36 districts from the period 2003–2008. With a strong reproductive health program and commitments from non-governmental organizations,† Zambia expanded in 2005–2006 and then maintained LLIN and IPTp distribution to antenatal clinics in all nine provinces. Similarly, Zambia expanded microscopy training, RDT use, availability of ACTs in all health facilities, and extended the use of antimalarial treatment through an increasing number of community health workers trained in malaria diagnosis and treatment; however, the evidence for these actions has been more descriptive than quantitative.^17^,^18^

#### Achieving high coverage.

**Figure 4.**
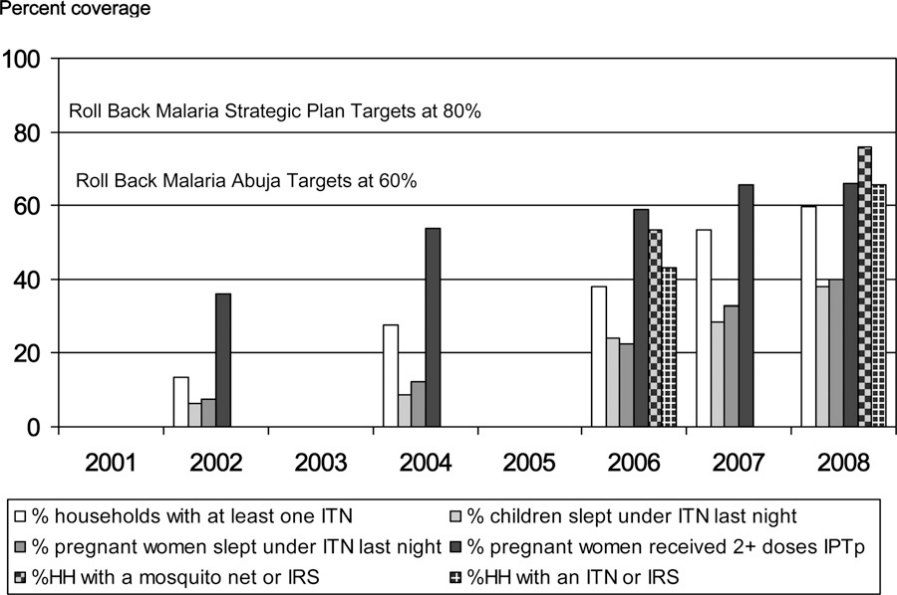
Malaria prevention intervention coverage progress in Zambia, 2002–2008.

Malaria control success is evident from population-based surveys monitoring intervention coverage rates at household and individual levels. From initial low coverage observed in the 2001/2-DHS, Zambia expanded malaria prevention coverage between 2004 and 2008. Figure 4 shows sequential national survey data demonstrating the increases in prevention coverage including ITN ownership, ITN use among children and pregnant women, and IPTp coverage in pregnancy. The summary figures from national surveys suggest that 68% (95% confidence interval [CI]: 64.2–72.4) of households have either one or more ITN or received IRS in the past year. This represents a 37% percent increase in household availability of effective malaria prevention nationally between 2006 and 2008 and a 5-fold increase between 2001/2 and 2008. The IRS coverage increased nearly 66% among districts targeted for spraying between 2006 and 2008, representing a substantial expansion of within-district coverage into rural, more malarious areas. Annual IRS operational coverage (the percentage of structures sprayed among those targeted each year) in each district has been consistently high since 2005—exceeding 85% in each target district. At this level of population coverage, one can expect that there is substantial community benefit even for those without an ITN or IRS.^19^,^20^

The proportion of febrile children less than 5 years of age who sought treatment at a facility or from a health care provider on the same or next day was similar in the MIS-2006 and MIS-2008, 60% and 64%, respectively. However, the rates of malaria treatment of febrile children less than 5 years of age decreased between the MIS-2006 and the MIS-2008 (52.8% [95% CI: 44.4–61.3] and 43.3% [95% CI: 39.0–47.6], respectively, an 18% reduction). This coincided with a slight increase in the use of ACTs from 9.6% (95% CI: 5.8–13.5) in 2006 to 12.7% (95% CI: 9.3–16.2) in 2008 and an expansion in the distribution of the reported use of malaria diagnostics (note that ~2 million RDTs were distributed to districts in 2008, Figure 2). Of note, when considering among children treated for malaria, ACT was used for 18.6% of treatments in 2006 and for 29% of treatments in 2008. This suggests that an increasing proportion of children with fever were tested for malaria leading to treatment of malaria only and not non-malarial fevers and that there has been some, but incomplete progress in using ACT for first line malaria therapy. Unfortunately, routine health information data does not include reporting from all districts on RDT use for testing febrile children, so full data are not available to assess national efforts in increasing use of diagnostics. Data on diagnostics use from Lusaka District (summarized elsewhere^21^) and data from several rural districts^18^ show substantial increases in use at least in some parts of the country. A summary of the changes in treatment practices between the Malaria Indicator Surveys of 2006 and 2008 is shown in Table 2.

#### Reduced disease and improved survival.

**Figure 5.**
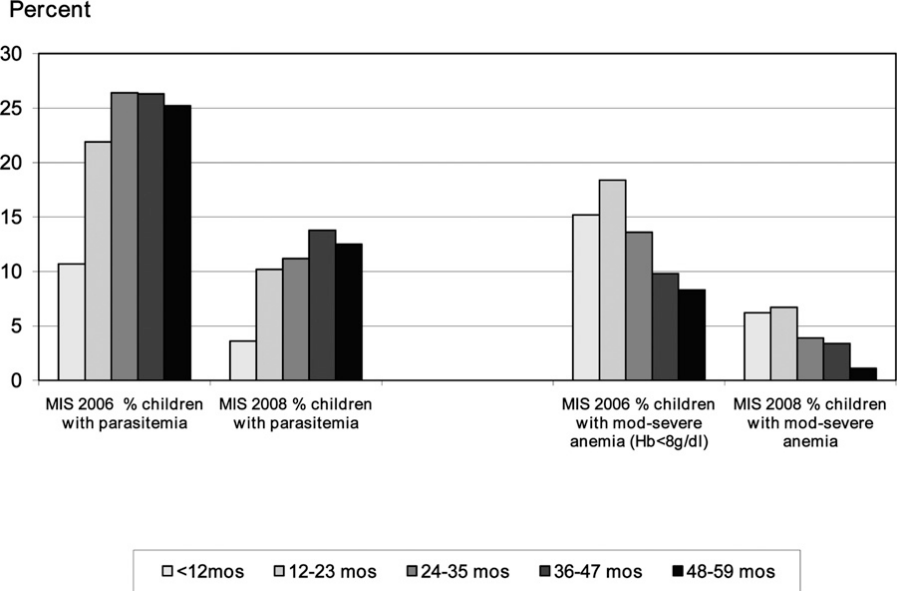
Child parasitemia and anemia rates by age group—findings from the Zambia 2006 and 2008 Malaria Indicator Surveys (MIS).

Evidence of health impact from malaria control in Zambia is mounting, especially among young children. Compared with MIS-2006, the MIS-2008, conducted at the same time of year, showed that malaria parasite prevalence in children less than 5 years of age was reduced by 53% (from 21.8% in 2006 to 10.2% in 2008) and moderate-severe anemia (hemoglobin < 8 gm/dL) in these children was reduced by 69% (from 13.8% in 2006 to 4.3% in 2008) and the reduction was seen in all age groups (Figure 5). On the basis of analysis from the MIS-2006, children in households with prevention in place (ITNs or IRS) had consistently lower rates of parasitemia and moderate-to-severe anemia.^22^

In addition to overall reductions in malaria among children less than 5 years of age between the MIS-2006 and MIS-2008, there was a 67% lower parasite prevalence in the first 12 months of life and a shift in the peak age of prevalence from the third year of life in 2006 to the fourth year of life in 2008 (Figure 5). This observation of delayed first infection and a longer parasite-free period in infancy is consistent with findings from the controlled trials of ITNs^23^ and is consistent with the observed improved infant survival also seen in those studies. Similarly, the substantial reduction in moderate-to-severe anemia rates occurred in all age groups among the children less than 5 years of age and this reduction is consistent with further improvements in infant and child survival.

National survey data show that all-cause child mortality has decreased by 29% from 168/1000 live births in DHS-2001/2 to 119/1000 live births in DHS-2007 (Table 2). Although there was a non-significant decrease in neonatal mortality (~8% decline), the substantial improvement is seen in post-neonatal infant mortality (from 28 to 365 days of life: 38% reduction) and in the 1- to 4-year-old children (36% reduction). Although this marked reduction in all-cause infant and child mortality may not be attributable only to improved malaria control, a review of other health programs suggests little or no change in coverage of interventions for other major causes of child mortality from recent nationally representative surveys (Table 3). Review of national rainfall data between 1980 and 2008 showed that dry years included 1992–1993 and 1994–1998, however rainfall was generally above the WASP baseline for most of the decade from 1999 to 2008 and this was particularly true for the interval from 2006 to 2008. Thus, it is unlikely that changes in rainfall patterns account for the reduced malaria during this recent interval.

#### Interventions have reached rural and poor populations.

**Figure 6.**
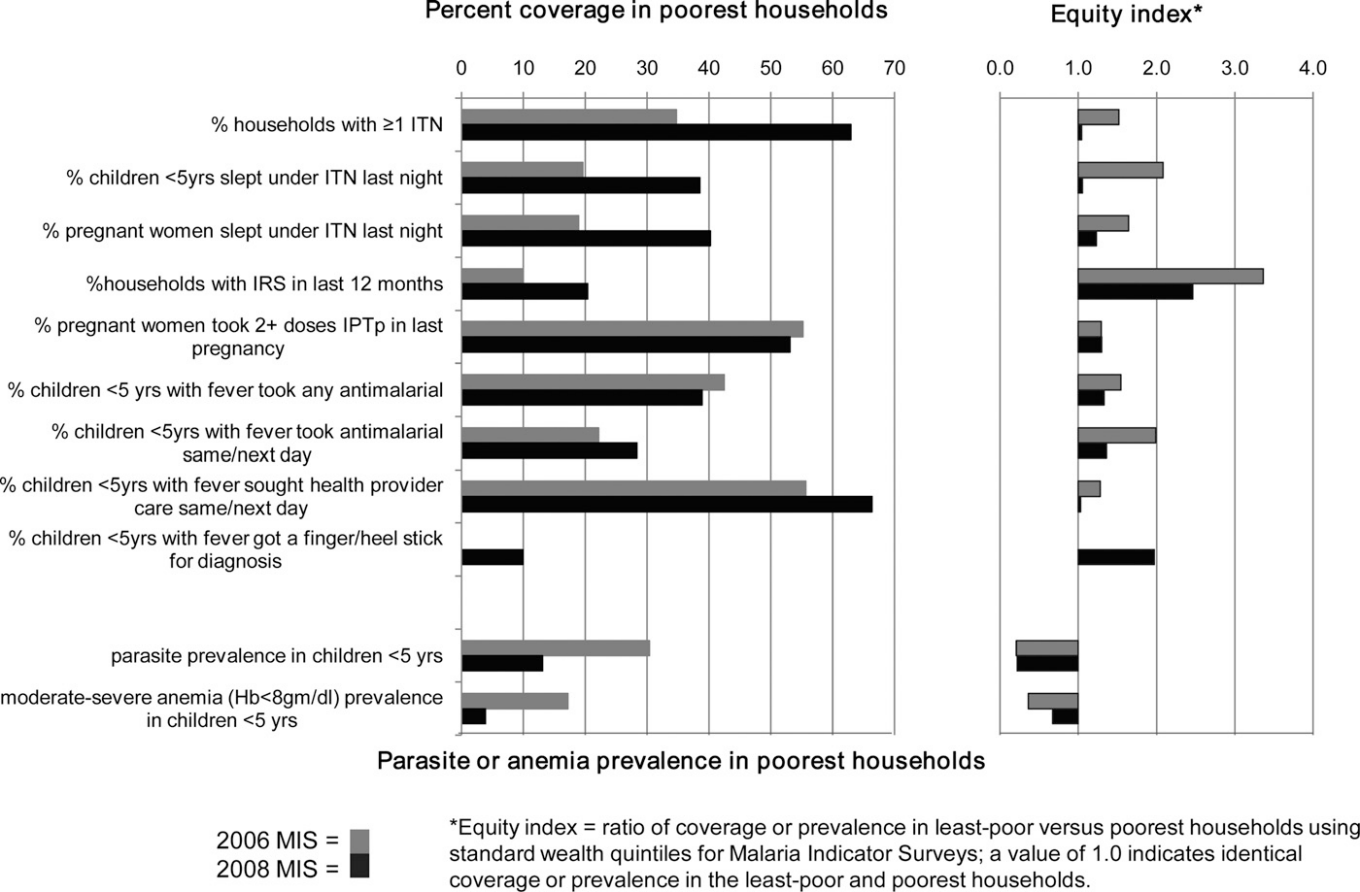
Changes in intervention coverage and prevalence of malaria and moderate-severe anemia in poorest households and changes in the equity ratio* between 2006 and 2008, Zambia.

The high national coverage rates extend to the poorer, more rural, and more malarious areas in Zambia. The coverage rates for the lowest wealth quintile households are shown in Figure 6 and equity ratios, defined as the wealth index quintile ratio between the least-poor households and the poorest households, of the key malaria indicators are presented. For each prevention and fever treatment indicator, the equity index has moved closer to 1.0 in the MIS-2008 compared with the MIS-2006; there has been no significant change for the IPTp indicators but these measures have an equity index close to 1.0 in both years; and for the morbidity indicators (where their equity index is less than 1.0—with parasitemia and anemia more common in poorer households), the anemia equity index has moved closer to 1.0. These findings suggest that progress has been made between 2006 and 2008 across the coverage indicators toward more equitable availability and use of malaria interventions and substantial and equitable reductions in malaria parasitemia and anemia.

## Discussion

In the years 2005 through 2008, Zambia systematically tracked malaria control program success in terms of attracting and using growing financial resources; increasing procurement and distribution of key commodities to homes and health facilities; increasing national coverage of interventions (particularly the prevention interventions of ITNs, IRS, and IPTp); reducing infection and illness; improving post-neonatal infant and child survival; and achieving equity of service delivery with concomitant disease reduction among the poorest and rural populations.

The documented progress is remarkable and to be celebrated by the country and those who contributed importantly in the work. The findings are consistent with what is known about the efficacy of the individual interventions.^24^–^28^ The documented reduction in all-cause child mortality (29% overall and ~37% in the post-neonatal infant and child periods) is substantial and perhaps larger than would be anticipated on the basis of malaria control alone. Although we looked for other disease prevention and control work that might be important contributing factors, none were readily apparent. Of note, although there was no change in childhood stunting and wasting, there was a 48% reduction in underweight children and this may have been due both to less repeated malaria infections and other factors of nutrient intake. There was an increase in the proportion of children with exclusive breast feeding in the first 6 months of life but this was coupled with a reduction in the proportion of children with continued breast feeding after 20 months of age. The rapid success seen in Zambia may be explained by the country's broadly semi-immune population which, when quickly covered with highly effective interventions that markedly decrease malaria transmission intensity, will show dramatic improvements within one malaria transmission season.

The consistency of the findings across the spectrum of receiving funding, documenting procurement and distribution of malaria prevention commodities, achieving high and equitable coverage, and showing national data on reduced morbidity and mortality makes a compelling story that malaria control is working. At the local level in communities and health facilities, the story is similar. The introduction of systems to record malaria outpatient cases, inpatient cases, and malaria deaths, with the presence of stable diagnostic capacity, have documented decreasing slide positivity rates in several facilities where reporting has been consistent and where interventions have been introduced.^21^ Similarly, the routine health information systems in districts where stable reporting has been maintained over time are showing substantial declines in both malaria infections and cases in malaria deaths.^29^ Thus, the national survey data and the local HMIS data are showing consistent progress in recent years.

Efforts in Zambia from 2005 through 2008 focused on scaling up malaria prevention services (ITNs and IRS for households, IPTp and ITNs for pregnant women), whereas curative services emphasized provision of improved diagnostics and quality of care for those attending facilities for malaria episodes.^30^ Despite being an early adopter of artemisinin-based combination therapy for treatment of malarial episodes,^31^ access to health care treatment services, especially in rural areas, remains challenging because of insufficient human resources for health^32^ and the difficulties in increasing quality care for febrile episodes.^11^,^13^ The Government of Zambia took bold steps to address universal access to health care by removing user fees in 2006 for rural areas,^33^ but many challenges remain to adequately address staffing requirements to meet established plans. More recently, the National Malaria Control Program embarked on an ambitious plan to train community health workers in malaria testing and treatment in response to calls for expanding access to treatment services.^34^,^35^

The nation of Zambia and its donor partners have invested extensively in malaria control and would undoubtedly like to see socio-economic progress along with the documented disease burden reduction. Although not a fully national effort, a recent review of data in the copper mining and sugar industries where company-supported malaria control efforts in the workforce and in the surrounding communities showed that this investment is improving production and is cost saving. Industry-collected data between 2001 and 2007 show that their investment in prevention has led to marked reduction in malaria cases in their workforce and families and improved work attendance, production-per-worker, and overall production for the plants. The data from 2006 accounting for money spent on malaria prevention and treatment and money recouped from fewer cases, fewer lost work hours and better production showed that Mopani Copper Mines saved US$295,718 and Zambia Sugar PLC saved US$550,379—saving approximately two dollars for every dollar invested in malaria prevention.^36^ Although it will take some time to assess links between malaria control and fully national economic benefits, the results of these industry studies are quite encouraging.

In the context of implementing a national program, there is no comparison population, other than historical, so this report can only be descriptive. However, information presented is from standard documented sources (DHS and MIS surveys, administrative and HMIS data, and industry case studies conducted with support from academic institutions) and the sum of these provides a broad consistency to the findings. This preliminary assessment of the dramatic drop in child mortality rates will be augmented by future national mortality studies allowing further exploration and analysis over time. The mortality data collected is “all-cause mortality” and not disease specific; thus, the further analysis will have limited ability to directly explore the contribution of malaria control to malaria-specific mortality decline. In addition, the DHS measures child mortality in the interval up to 5 years before the survey (e.g., 2007 survey measures mortality from 2003 through 2007), however this likely includes changes that might have occurred related to the scale up of malaria control interventions in the 2005–2007 interval. Although theoretically one could examine mortality rates in the 2003–2004 interval and compare them with the 2005–2007 interval, the DHS sample size precludes statistically relevant comparisons for these sub-analyses. Recognizing these limitations, the initial review of the DHS survey data in 2001/2 and 2007 suggests that the largest change in disease control has been the rapid scale up of malaria interventions and this likely has contributed importantly to the overall improved child survival.

Zambia is now moving toward completing the final steps for achieving and sustaining high national coverage rates of malaria control interventions to further reduce the remaining morbidity, mortality, and transmission. Much of the success in reducing illness and death has already been achieved and one can anticipate that further declines in illness and death rates will be only modest in comparison to what has already been achieved. Thus, future success will be seen in holding morbidity and mortality at these new low levels and any future improvements will need to be compared with the pre-scale-up mortality estimates from the DHS 2001/2. As Zambia expands program work to further reduce malaria transmission, the program will need to consider specific measures of transmission that can be tracked over time to understand success in the future.

## Conclusion

Zambia, a fully malaria-endemic country, has achieved substantial and rapid success in reducing the malaria burden across the nation. The findings here show that the success in malaria control can be systematically tracked across policy improvements, financial investments, actions of procurement and distribution, increases in intervention coverage, reductions in illness and death, with improvements across all populations including the poor and rural areas, and achieving economic benefits. Sustaining these gains will be extremely important, but this next sustaining phase will not be accompanied by huge new reductions in illness and death as the current much lower levels suggest that the remaining child morbidity and mortality may be the result of other causes. This could lead to complacency in malaria control efforts within the country and from the external partners that have greatly aided this effort. Decision makers could move to support new and different priorities; if the complacency or shifting focus leads to undoing current gains against malaria, high malaria morbidity and mortality rates can be expected to return as has occurred elsewhere in recent times.^37^–^39^ Thus, the critical next step is to sustain the current gains and further reduce malaria transmission. The scale up phase in malaria control was initially thought to be quite challenging; it may be the easy part as countries and partners target sustained malaria control and, ultimately, malaria elimination.

For Zambia to take additional steps to reduce the remaining malaria transmission, continued resources and new work will be required. As the infection and disease become more focal, community techniques to map malaria cases and transmission and an approach of testing and treating the remaining infected population will be required.^40^ This approach is under consideration in an initial set of villages. Only by anticipating the next steps in sustained control, presumably the same steps that will be ultimately needed for elimination, will Zambia (and other countries) be able to achieve final malaria control success.

## Figures and Tables

**Table 1:** National Malaria Strategic Plan targets in Zambia*

National Malaria Strategic Plan 2006–2011	
	Target
ITN coverage target	> 80% of HH with average of 3 ITN/HH
IRS coverage target	> 85% coverage of eligible HH in 15 target districts
IPTp coverage target	> 80% of pregnant women receiving ≥ 2 doses IPTp
Target for ITN use in pregnant women	> 80% of pregnant women sleeping under ITN or in a house with IRS
Target for ITN use in children < 5 years of age	> 80% of children < 5 sleeping under ITN or in a house with IRS
Target for PECM	> 80% of sick persons treated with effective antimalarial within 24 hours of onset

* RBM = Roll Back Malaria; ITN = insecticide-treated mosquito nets; HH = household; IRS = indoor residual spraying; IPTp = intermittent preventive treatment during pregnancy; PECM = prompt effective case management (defined as treatment with recommended antimalarial drug within 24 hours of illness onset).

**Table 2:** Intervention coverage, parasitemia, and anemia changes between MIS-2006 and MIS-2008*

Indicator	MIS-2006	MIS-2008	% Change
Coverage of intervention			
Among households (HHs):	*N =* 2,881	*N =* 4,405	
HHs with ≥ 1 ITN	37.8% (95% CI: 33.6–42.0)	62.3% (95% CI: 58.2–66.5)	↑ 65%
HHs with ≥ 2 ITNs	14.4% (95% CI: 11.6–17.2)	30.9% (95% CI: 27.6–34.2)	↑ 115%
HHs with IRS in the last 12 months among IRS-targeted districts	25.8% (95% CI: 17.6–34.0)	42.7% (95% CI: 35.0–50.5)	↑ 66%
HHs with ≥ 1 ITN or IRS in last 12 months	43.2% (95% CI: 38.7–47.8)	68.3% (95% CI: 64.2–72.4)	↑ 58%
Among pregnant women (PW)†:	*N* = 317	*N* = 416	
PW slept under ITN last night	24.5% (95% CI: 18.9–30.1)	43.2% (95% CI: 36.6–69.3)	↑ 76%
PW‡ took any IPTp	71.6% (95% CI: 68.1–75.1)	80.0% (95% CI: 77.3–82.8)	↑ 12%
PW‡ took 2 + doses IPTp	58.8% (95% CI: 55.3–62.1)	66.1% (95% CI: 62.9–69.3)	↑ 12%
Among children under age five:	*N* = 2,539	*N* = 3,866	
Child slept under ITN last night	24.3% (95% CI: 21.2–27.5)	41.1% (95% CI: 37.2–45.0)	↑ 69%
Child with fever§ received any antimalarial treatment	52.8% (95% CI: 44.4–61.3)	43.3% (95% CI: 39.0–47.6)	↓ 18%
Child with fever§ received antimalarial treatment on same/next day	31.0% (95% CI: 24.2–39.4)	28.9% (95% CI: 24.6–33.3)	↓ 9%
Child with fever§ had finger/heel prick (diagnostic used)	na	10.9% (95% CI: 7.5–14.3)	–
Child with fever received Coartem	9.6% (95% CI: 5.8–13.5)	12.7% (95% CI: 9.3–16.2)	↑ 32%
Health outcomes among children < age 5			
Malaria parasite prevalence	21.8% (95% CI: 17.2–26.5)	10.2% (95% CI: 7.7–12.6)	↓ 53%
Urban	6.4% (95% CI: 2.7–10.2)	4.3% (95% CI: 2.2–6.3)	↓ 33%
Rural	26.9% (95% CI: 20.9–32.9)	12.4% (95% CI: 9.2–15.7)	↓ 54%
Mean hemoglobin (g/dL)	9.97 (95% CI: 9.80–10.14)	10.91 (95% CI: 10.81–11.0)	↑ 9%
Severe anemia prevalence (Hb < 8.0 g/dL)	13.8% (95% CI: 11.1–16.6)	4.3% (95% CI: 3.3–5.3)	↓ 69%

* HH = household; CI = confidence interval; ITN = insecticide-treated mosquito nets; IPTp = intermittent preventive treatment in pregnancy; ns = not available.

† Women who reported themselves as currently pregnant.

‡ For preventive treatment during pregnancy, estimates are based on the last birth among women during the previous 5 years. For MIS 2006, *N* = 1,572 and for MIS 2008 *N* = 2,391.

§ Estimates of children with fever are based on *N* = 363 for MIS 2006 and *N* = 843 for MIS 2008.

**Table 3:** Information related to major child health program coverage that may have contributed to reductions in all-cause child mortality between the DHS-2001/2 and the DHS-2007

Indicator	2001/2 DHS	2007 DHS	Percent change (%)
Mortality rates*			
Infant mortality (0–11 months)	95	70	↓ 26
Neonatal mortality (< 1 month)	37	34	↓ 8
Post-neonatal mortality (1–11 months)	58	36	↓ 38
Child mortality (1–4 yrs)	81	52	↓ 36
Under 5 mortality (0–5 years)	168	119	↓ 29
Coverage of interventions among children			
Percent stunted (children under 5 years)	46.8	45.4	↓ 3
Percent wasted (children under 5 years)	5.0	5.2	↑ 4
Percent underweight (children under 5 years)	28.1	14.6	↓ 48
Percent of youngest children under 6 months who are exclusively breastfed	40.1	60.9	↑ 52
Percent of children age 12–15 months still breastfeeding	96.8	93.8	↓ 4
Percent of children age 20–23 months still breastfeeding	55.5	41.7	↓ 25
Percent of children age 12–23 months with BCG vaccination	94.0	92.3	↓ 2
Percent of children age 12–23 months with at least 3 polio vaccinations	80.2	77.0	↓ 4
Percent of children age 12–23 months with measles vaccination	84.4	84.9	↑ 1
Percent of children age 0–59 months with diarrhea in the 2 weeks preceding the survey who received oral rehydration salts (ORS) or recommended home fluids	66.9	66.8	↔ 0
Percent of children age 0–59 months with acute respiratory infection (ARI) in the 2 weeks preceding the survey who were taken to a health provider	69.1	68.2	↓ 2
Percentage of households with at least one ITN	13.6	53.3	↑ 292
Percentage of children 0–59 months who slept under an ITN on the previous night	9.8	28.5	↑ 192
Percentage of pregnant women 15–49 who slept under an ITN on the previous night	7.9	32.7	↑ 314
Percent of children age 0–59 months with a fever in the 2 weeks preceding the survey who took an anti-malarial drug	51.9	38.4	↓ 27
Percent of children age 0–59 months with a fever in the 2 weeks preceding the survey who took an anti-malarial drug the same day/next day after developing fever	36.8	20.5	↓ 44

* Mortality calculated as deaths per 1,000 live births except for child mortality, which is calculated as deaths per 1,000 children surviving to 12 months of age.

## Footnotes

† The Churches Association of Zambia (CHAZ), a principal recipient of malaria funding from the GF, plays an important role in coordinating health care service delivery, including antenatal services, among an extensive network of religious or mission-based health care facilities throughout Zambia. Population Sciences International (PSI) and their local affiliate, Society for Family Health, in Zambia has external donor support and works closely with the Reproductive Health Unit in the Zambia MOH to deliver malaria prevention through ITNs to pregnant women and their children (http://www.psi.org/Where_We_Work/zambia.html).
